# The impact of flattening-filter-free beam technology on 3D conformal RT

**DOI:** 10.1186/1748-717X-8-133

**Published:** 2013-05-31

**Authors:** Matthias Kretschmer, Marcello Sabatino, Arne Blechschmidt, Sebastian Heyden, Bernd Grünberg, Florian Würschmidt

**Affiliations:** 1 Department of Radiation Therapy and Radiooncology, Radiologische Allianz Hamburg, Hamburg, Germany; 2 Department of Radiation Therapy and Radiooncology, Klinikum Görlitz, Görlitz, Germany

**Keywords:** Flattening-filter-free, Forward planning, 3D-CRT, Planning study

## Abstract

**Background:**

The removal of the flattening filter (FF) leads to non-uniform fluence distribution with a considerable increase in dose rate. It is possible to adapt FFF beams (flattening-filter-free) in 3D conformal radiation therapy (3D CRT) by using field in field techniques (FiF). The aim of this retrospective study is to clarify whether the quality of 3D CRT plans is influenced by the use of FFF beams.

**Method:**

This study includes a total of 52 CT studies of RT locations that occur frequently in clinical practice. Dose volume targets were provided for the PTV of breast (n=13), neurocranium (n=11), lung (n=7), bone metastasis (n=10) and prostate (n=11) in line with ICRU report 50/62. 3D CRT planning was carried out using FiF methods. Two clinically utilized photon energies are used for a Siemens ARTISTE linear accelerator in FFF mode at 7MV_FFF_ and 11MV_FFF_ as well as in FF mode at 6MV_FF_ and 10MV_FF_. The plan quality in relation to the PTV coverage, OAR (organs at risk) and low dose burden as well as the 2D dosimetric verification is compared with FF plans.

**Results:**

No significant differences were found between FFF and FF plans in the mean dose for the PTV of breast, lung, spine metastasis and prostate. The low dose parameters V5Gy and V10Gy display significant differences for FFF and FF plans in some subgroups. The DVH analysis of the OAR revealed some significant differences. Significantly more fields (1.9 – 4.5) were necessary in the use of FFF beams for each location (p<0.0001) in order to achieve PTV coverage. All the tested groups displayed significant increases (1.3 – 2.2 times) in the average number of necessary MU with the use of FFF beams (p<0.001).

**Conclusions:**

This study has shown that the exclusive use of a linear accelerator in FFF mode is feasible in 3D CRT. It was possible to realize RT plans in comparable quality in typical cases of clinical radiotherapy. The 2D dosimetric validation of the modulated fields verified the dose calculation and thus the correct reproduction of the characteristic FFF parameters in the planning system that was used.

## Background

Traditionally, the flattening filter (FF) in the X-ray beam path of a linear accelerator produces an almost uniform fluence over a collimated field. This is particularly advantageous for 3D conformal radiation therapy (3D CRT) for practical reasons.

The removal of the flattening filter leads to a radially decreasing fluence distribution and thus to inhomogeneous dose distributions. The advantage of this is its positive influence on the peripheral dose through reduced head scatter and MLC leakage [1], as well as a considerable increase in the dose rate, which has a beneficial effect on modern therapy methods.

Besides improved shielding in the treatment head Hall et al. in 2006 [2] also suggested the use of secondary jaws to track the MLC and removal of the flattening filter as a source of scattered radiation with fluence-modulated RT. The disadvantage of a non-uniform, conical fluence distribution can be taken into account with intensity modulated radiation therapy (IMRT) in the optimization algorithm. Recent studies have shown the feasibility of the use of FFF beams for IMRT and stereotactic body radiation therapy (SBRT) [3-5].

It has also been concluded that the decreased variation in scatter factors and beam quality along the field will simplify dose calculation [6]. It is often necessary to resort to field in field techniques (FiF), which are often also termed forward IMRT techniques, in order to achieve better conformity for the PTV in 3D CRT planning. Additional fields in one angle of incidence (multistatic field) can be used to adapt dose distribution optimally to the anatomy of the patient without the need for a wedge. Several studies for various RT locations have shown that a beneficial dose distribution can be achieved with this method in relation to homogeneity and conformity [7-9]. It is also possible to adapt FFF beams in 3D CRT by using this field in field technique.

The aim of this retrospective study is to clarify whether the quality of 3D conformal RT plans is influenced by the use of FFF beams. Large-volume disease sites that are routinely treated with RT, including both homogeneous and heterogeneous locations, were evaluated in this study. The plan quality in relation to target volume coverage, organs at risk and low-dose exposure is compared with conventional FF plans along with dosimetric verification of dose delivery at the linear accelerator. Finally the question shall be answered whether it is possible to manage clinical routine 3D CRT cases with FFF.

## Methods

### Patient population, dose prescription and target delineation

Patient studies were acquired from clinical practice at Radiologische Allianz Hamburg in the period between April 2011 and June 2011. In order to study as many influences as possible in the use of FFF fields, the patient cohort was combined taking the following criteria into account: frequency of the RT location in clinical practice, PTV in homogeneous and highly inhomogeneous environments, PTV that is deep or close to the surface and high volume range between the different tumor entities. Table 1 shows the selected RT locations and the inclusion criteria in this retrospective planning study. For breasts the PTV included the entire left or right mammary gland without parasternal, axial or supraclavicular lymph nodes. The PTV for lung tumors is formed by the mediastinal lymph nodes including the left or right hilus. The PTV for neurocranium RT included the entire brain. The PTV for spine metastasis covered a maximum of four vertebrae including a safety margin. For RT locations of the prostate the PTV included either the prostate with the seminal vesicles or the prostatic bed after prostatectomy including a safety margin. For practical reasons dose prescriptions for all locations were considered for the main series without possible boost volumes. Breast, neurocranium, lung, bone metastasis and prostate dose volume constraints are provided for the PTV in line with ICRU report 50/62.

**Table 1:** Summary of inclusion criteria for the studied RT locations with clinically oriented dose prescription and planning objectives for the PTV

**Tumor site**	**Study criteria**	**Prescription** [**Gy**]	**Dose**/**fx** [**Gy**]	**PTV constraints**	**n**	**Comments**
Breast	Whole Breast, without supraclavicular LN	50.0	2.0	V95%>90%, D2%<107%	13	
Lung	Mediastinum ± hilus, without supraclavicular LN	50.0	2.0	V95%>95%, D2%<107%	7	
Neurocranium	Whole brain	30.0	3.0	V95%>99%, D2%<107%	11	
Bone Metastasis	Spine locations with max. 4 vertebrae	37.5	2.5	V95%>99%, D2%<107%	10	C=3, TH=5, L=2
Prostate	Prostatic bed/ prostate	66.0(Prostatic bed)	2.0	V95%>99%, D2%<107%	11	

*Vx* volume percentage receiving at least x % of the prescribed dose, *Dx *% dose received by at least x % of the volume, Abbreviations: *n* number of patients, *LN* lymph nodes, *C* cervical vertebra, *TH* thoracic vertebra, *L* lumbar vertebra.

In line with ICRU report 50/62 the target dose of V95%>99% (99% of the PTV receiving at least 95% of the dose) and D2%<107% (2% of the PTV receiving a maximum 107% of the dose) was prescribed for the PTV for neurocranium, bone metastasis and prostate. Due to the build up effect, V95%>90% was prescribed for PTV breast and V95%>95% for PTV lung. Table 2 shows the OAR contoured for the relevant RT location. The dose constraints conform to the QUANTEC data [10] and should be kept as low as possible during the planning process. Healthy tissue was defined as the outer contour of the patient excluding the PTV. A total of 52 patient studies were included. The PTV definition was carried out by four radiation oncologists on CT scans (Somatom Definition AS20, Siemens AG, Erlangen, Germany) with 2 mm slice thickness.

**Table 2:** **Summary of the plan setup**, **contoured OAR**, **photon energy and planning method used for the relevant RT locations**

**Tumor site**	**Relevant study OAR**	**Field setup**	**Energy** [**MV**] (**FF**/**FFF**)	**Technique**
Breast	Contralateral lung	Tangential field setup	6 / 7	FiF
	Ipsilateral lung		10 / 11	
	Heart			
	Healthy tissue			
Lung	Contralateral lung	AP, PA, LO	6 / 7	FiF
	Ipsilateral lung		10 / 11	
	Heart			
	Myelon			
	Healthy tissue			
Neurocranium	Right eye	Lateral opposing	10 / 11	FiF
	Left eye			
	Healthy tissue			
Bone Metastasis	Myelon	AP, PA, RPO, LPO	6 / 7	FiF and virtual wedges for FF, FiF for FFF
	Healthy tissue		10 / 11	
Prostate	Bladder	AP, RLO, LLO, RPO, LPO	10 / 11	FiF
	Rectum			

	Healthy tissue			

Abbreviations: *R*/*L PO* right/left posterior oblique, *LPO* left posterior oblique, *AP* anterior-posterior, *PA* posterior-anterior, *L*/*R LO* left/right lateral oblique, *FiF* field in field.

### Treatment planning

Treatment planning was carried out using version 4.1 of MasterPlan (Nucletron/ELEKTA, Veenendaal, Netherlands) with an enhanced collapse cone calculation algorithm (eCC). Studies by Kragl et al. substantiate at least equivalent dose calculation accuracy between FF and FFF beams [11]. Two clinically utilized photon energies from ARTISTE linear accelerators (Siemens AG, Erlangen, Germany) were used at Klinikum Görlitz (7MV_FFF_ and 11MV_FFF_) and Radiologische Allianz Hamburg (6MV_FF_ and 10MV_FF_). Further details and beam characteristics were investigated by Dzierma et al. [12]. Both LINACs are equipped with a 160 leaf MLC. The leaf width is 5 mm projected to the isocenter. Interdigitation for all leaves is possible. Further details and dosimetric characteristics were investigated by Tacke et al. [13]. Except the flattening filter, the beamline for FFF mode (7MV_FFF_ or 11MV_FFF_) is the same as for 6MV or 10MV. The dose rate in FFF mode is 2000 MU/min regardless of the selected photon energy. In FF mode 300 MU/min are provided for 6MV and 500 MU/min are provided for 10MV. The deviation of the dose calibration in the isocenter at SSD=90 cm and a field size of 10 × 10 cm^2^ was under 1% for both energy pairs (6MV_FF_ 7MV_FFF_ and 10MV_FF_ 11MV_FFF_).

**Figure 1:**
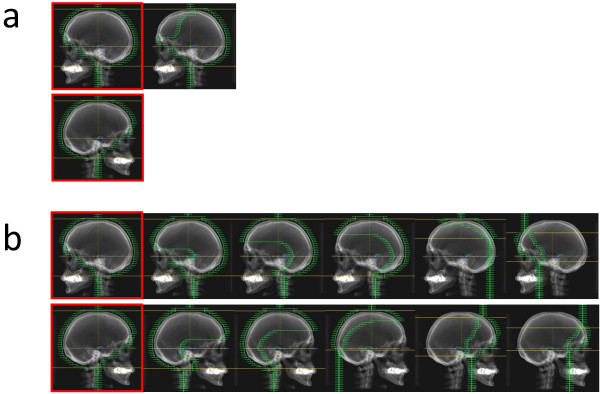
**Principle of field in field technique.** Representation of the field in field method (FiF) using the example of a neurocranium RT. The diagrams outlined in red show the initial MLC fields for one beam direction that are identical for FF and FFF planning. The upper row (**a**) shows the fields at 10MV that are necessary to achieve the planning objectives. The additional field serves to block off overdosed areas. The lower row (**b**) shows the required additional fields because of the significant drop in radial intensity at 11MV FFF. In this case ten additional fields are required to compensate the under-dosing in order to achieve the planning objectives.

Treatment planning was carried out by four experienced medical physicists using FiF methods. MLC field copies were created of the initial direction of the beams. Those subfields were manually shaped with the aim of minimizing hot/cold spots resulting from the initial beam set up (Figure 1). A FF plan and a FFF plan were created for each patient study. The isocenter was placed in the center of the volume of the PTV. The FF plan approved by the radiation oncologist served as a clinical reference here. The initial beam directions and the dose prescription in the reference FF plans were used to create the FFF plans. 6MV_FF_ was replaced with 7MV_FFF_ and 10MV_FF_ with 11MV_FFF_.

FFF plans were not used clinically on patients at any time. Use of virtual wedges was also permitted for RT location in bone metastasis (FF). Table 2 shows the plan set up that was used for the relevant RT location.

### Statistical analysis

A test of significance is required in order to quantify the differences between parameters in FF and FFF plans. Since the DVH analysis was collected for the same patient collective, a two-sided, paired student t-test was used. Statistical significance was defined for p-values below 0.01.

### Evaluation methods

The plans were compared and analyzed using DVH information. Volume, mean dose, the volume that receives 95% or 107% of the prescribed dose (V95% and V107%) and the homogeneity index HI were determined for all PTV. The homogeneity index (*HI*= [D2%-D98%] / D_prescription_) reflects how uniform the dose is in the PTV. A smaller *HI* indicates a more homogeneous dose distribution. The PTV was retracted 3 mm from the outline in the case of breast plans because dose calculation algorithms have difficulties modelling the build-up effect. The modified PTV was used for further analysis. Low-dose exposure of healthy tissue was reported as the volume that receives 5 Gy (V5Gy) and 10 Gy (V10Gy). The healthy tissue was defined as external contour minus the PTV.

For breast and lung plans, mean dose and V20Gy was taken for the ipsilateral lung and the mean dose was taken for the contralateral lung and the heart. For lung cases the maximum dose of the OAR myelon over the parameter D2% (dose received by at least 2% of the volume) was also taken. For the neurocranium plans the mean dose was determined for both eyes. No OAR analysis was carried out for spine metastasis because of the varying positions inside the spine (cervical vertebrae n=3, thoracal vertebrae n=5, lumbar vertebrae n=2). In prostate plans the mean dose and V50Gy were determined for bladder and rectum. The number of fields required to achieve the planning objectives and the cumulated MU were also recorded for all plans. All results for this study are reported as averages of the investigated RT location and the appropriate standard deviation.

### Dosimetric verification

Six FFF plans for each of the studied tumor locations (n=36) were prepared on the ARTISTE in Görlitz for 2D dose measurements with the Octavius phantom and 729 Array (PTW, Freiburg, Germany). All fields in one beam direction of a plan were combined (multistatic field) and mapped under a gantry angle of 0° while retaining the monitor units on the scanned Octavius phantom in order to generate RT dose cubes in MasterPlan. Overall 96 modulated fields were created and compared with array measurements using the gamma criterion [14]. The percentage share of γ <1 with 3 mm DTA (distance-to-agree) and 3% dose difference for doses over 5% was determined in relation to the dose maximum. No dosimetric analysis took place of the FF plans on the ARTISTE linear accelerator in Hamburg.

In order to measure the performance these plans were irradiated as an automatic sequence and the time measured between the first field and the end of the last field. The beam application took place in Görlitz under the control of MOSAIQ (version 2.00) verification system and in Hamburg using SYNGO Therapist (version 4.1) and LANTIS (version 6.1) verification system.

## Results

### Dose-coverage for PTV

Table 3 shows the results of the DVH analysis for the relevant PTV. No significant differences were found between FFF and FF plans in the mean dose for breast, lung, spine metastasis and prostate. For the neurocranium group a significant mean dose difference (p=0.009) was found of 30.3 ± 0.2 Gy (FFF) versus 30.9 ± 0.3 Gy (FF). It was always possible to achieve the prescribed dose for both types of treatment techniques. The specific requirements for the minimal dose coverage V95% were achieved for all groups with the exception of the spine metastasis group. On average, FFF plans were slightly under-dosed at 97.7 ± 2.6% vs. 98.3 ± 3.3% (FF). However, the under-dose is statistically not relevant (p=0.230). Significant differences in the PTV homogeneity HI could be seen for breast (p<0.001) at 0.15 ± 0.01 (FF) vs. 0.17 ± 0.01 (FFF), and for neurocranium (p<0.001) at 0.06 ± 0.01 (FF) vs. 0.08 ± 0.01 (FFF).

**Table 3:** DVH analysis for PTV and healthy tissue for treatment plans created with FF and FFF beams

**Parameter**	**Breast**	**Neurocranium**	**Lung PTV**	**Spine metastasis**	**Prostate**
n	13	11	7	10	11
Volume [cm^3^]	922.7 ± 239.4	1329.3 ± 109.3	501.9 ± 318.0	175.7 ± 79.6	211.3 ± 94.9
Mean+SD [Gy]	50.2 ± 0.3	30.9 ± 0.3	50.3 ± 0.2	37.9 ± 0.4	66.6 ± 0.6
	**50.2 ± 0.4**	**30.3 ± 0.2**	**50.5 ± 0.4**	**38.0 ± 0.3**	**66.5 ± 0.6**
p	0.495	0.000	0.408	0.230	0.622
V95 [%]	89.8 ± 1.6	99.9 ± 0.0	95.4 ± 3.3	98.3 ± 3.3	99.6 ± 0.3
	**89.1 ± 1.8**	**99.4 ± 0.3**	**94.3 ± 2.4**	**97.7 ± 2.6**	**99.2 ± 0.3**
p	0.166	0.000	0.120	0.280	0.027
V107 [%]	0.0 ± 0.0	0.0 ± 0.0	0.0 ± 0.0	0.0 ± 0.0	0.0 ± 0.0
	**0.0 ± 0.0**	**0.0 ± 0.0**	**0.1 ± 0.3**	**0.0 ± 0.0**	**0.0 ± 0.0**
p	0.166	0.000	0.234	0.133	0.459
HI	0.15 ± 0.0	0.06 ± 0.0	0.12 ± 0.0	0.10 ± 0.0	0.08 ± 0.0
	**0.17 ± 0.0**	**0.08 ± 0.0**	**0.13 ± 0.0**	**0.11 ± 0.0**	**0.09 ± 0.0**
p	0.000	0.000	0.151	0.027	0.023
			**Healthy tissue**		
V5Gy [%]	7.8 ± 1.1	54.7 ± 10.0	27.6 ± 9.5	10.4 ± 2.4	19.8 ± 8.3
	**7.7 ± 1.1**	**54.3 ± 10.0**	**27.7 ± 8.0**	**10.2 ± 2.4**	**20.0 ± 8.2**
p	0.006	0.000	0.921	0.341	0.639
V10Gy [%]	6.2 ± 1.0	50.8 ± 9.4	20.0 ± 7.3	6.2 ± 1.7	14.4 ± 6.7
	**6.2 ± 1.0**	**50.4 ± 9.4**	**19.6 ± 6.4**	**6.1 ± 1.7**	**14.6 ± 6.5**
p	0.963	0.000	0.553	0.202	0.293

FFF results are in bold. VxGy: volume receiving at least x Gy. Vx: volume receiving at least x % of the prescribed dose. Dx %: dose received by at least x % of the volume. Statistical significance is defined for p < 0.01.

### Organs at risk and low dose exposure

Table 3 shows the results of the DVH analysis for the healthy tissue. The low dose parameters V5Gy and V10Gy show very similar and mostly non-significantly differing results for FFF and FF plans. V5Gy is significantly lower (p=0.006) in the breast group for FFF at 7.7 ± 1.1% vs. 7.8 ± 1.1% (FF).

In Tables 4 and 5 the results are shown for DVH analysis of the OAR of the studied locations. In the breast group the mean ipsilateral lung exposure was significantly lower (p=0.001) for the FFF at 8.9 ± 1.5Gy vs. 9.1 ± 1.5Gy (FF). Non-significant and comparable results were found for V20Gy. Contralateral lung sections receive an average of 0.5 ± 0.0Gy (FFF) vs. 0.6 ± 0.1Gy (FF) (p=0.011). Significant differences were found in the mean eye dose for neurocranium plans: for the left eye, 8.3 ± 2.4Gy (FF) vs. 7.3 ± 2.4Gy (FFF) (p<0.001) and for the right eye 9.1 ± 2.4Gy (FF) vs. 8.1 ± 2.4Gy (FFF) (p<0.001) were observed. The lung group does not show any significant differences in the mean lung dose or V20Gy for ipsilateral and contralateral lung. It was possible to determine an average increase in mean heart dose of 0.8Gy (11.8 ± 5.6Gy for FFF vs. 11.0 ± 5.5Gy for FF) (p=0.011). The maximum dose D2% in the myelon is not significantly higher (p=0.697) with 29.0 ± 5.9Gy for FF vs. 29.4 ± 7.7Gy for FFF. Because of varying positions within the spine (C=3, TH=5, L=2) no further OAR analysis (except the myelon) was undertaken in the spine metastasis group. The D2% to the myelon in this group was significantly higher in FFF plans (39.3 ± 0.5Gy vs. 38.4 ± 0.4Gy for FF, p=0.001). In the prostate group the mean and the V50Gy dose for rectum and bladder were increased in FFF plans (Table 5). The increases were in the range of 0.8Gy – 1.5Gy (p>0.01).

**Table 4:** **DVH analysis for OAR of the groups breast**, **lung and neurocranium for treatment plans created with FF and FFF beams**

**Parameter**	**Breast**	**Neurocranium**	**Lung**
		**OAR**	
n	13	11	7
		**Left eye**	
Mean+SD [Gy]	-	8.3 ± 2.4	-
	-	**7.3 ± 2.4**	-
p	-	0.000	-
		**Right eye**	
Mean+SD [Gy]	-	9.1 ± 2.4	-
	-	**8.1 ± 2.4**	-
p	-	0.000	-
		**Ipsilateral lung**	
Volume [cm^3^] Mean+SD [Gy]	1791.2 ± 313.6	-	2018.6 ± 319.2
	9.1 ± 1.5	-	17.6 ± 2.8
	**8.9 ± 1.5**	-	**17.5 ± 3.2**
p	0.000	-	0.700
V20Gy [%]	17.7 ± 3.5	-	43.0 ± 7.1
	**17.6 ± 3.**4	-	**40.6 ± 9.4**
p	0.042	-	0.189
		**Contralateral lung**	
Volume [cm^3^]	1660.2 ± 394.7	-	2479.0 ± 806.2
Mean+SD [Gy]	0.6 ± 0.1	-	6.8 ± 4.6
	**0.5 ± 0.0**	-	**6.8 ± 3.5**
p	0.011	-	0.903
		**Heart**	
Volume [cm^3^]	555.1 ± 280.4	-	-
Mean+SD [Gy]	2.9 ± 2.0	-	11.0 ± 5.5
	**2.8 ± 2.1**	-	**11.8 ± 5.6**
p	0.018	-	0.011

FFF results are in bold. *VxGy* volume receiving at least x Gy. *Vx* volume receiving at least x % of the prescribed dose, *Dx *% dose received by at least x % of the volume, Statistical significance is defined for p < 0.01.

**Table 5:** **DVH analysis for OAR of the groups lung**, **spine metastasis and prostate for treatment plans created with FF and FFF beams**

**Parameter**	**Lung**	**Spine metastasis**	**Prostate**
	**OAR**		
n	7	10	11
	**Rectum**		
Volume [cm^3^]	-	-	75.8 ± 47.2
Mean+SD [Gy]	-	-	48.6 ± 10.7
	-	-	**49.8 ± 10.7**
p	-	-	0.020
V50Gy [%]	-	-	52.2 ± 19.5
	-	-	**56.4 ± 20.8**
p	-	-	0.024
	**Bladder**		
Volume [cm^3^]	-	-	227.8 ± 128.5
Mean+SD [Gy]	-	-	29.5 ± 13.7
	-	-	**31.0 ± 14.2**
p	-	-	0.019
V50Gy [%]	-	-	29.8 ± 20.9
	-	-	**31.6 ± 21.2**
p	-	-	0.041
	**Myelon**		
D2% [Gy]	29.0 ± 5.9	38.4 ± 0.4	-
	**29.4 ± 7.7**	**39.3 ± 0.5**	-
p	0.697	0.001	-

FFF results are in bold. VxGy: volume receiving at least x Gy. Vx: volume receiving at least x % of the prescribed dose. Dx %: dose received by at least x% of the volume. Statistical significance is defined for p < 0.01.

### Relative dose distributions

**Figure 2:**
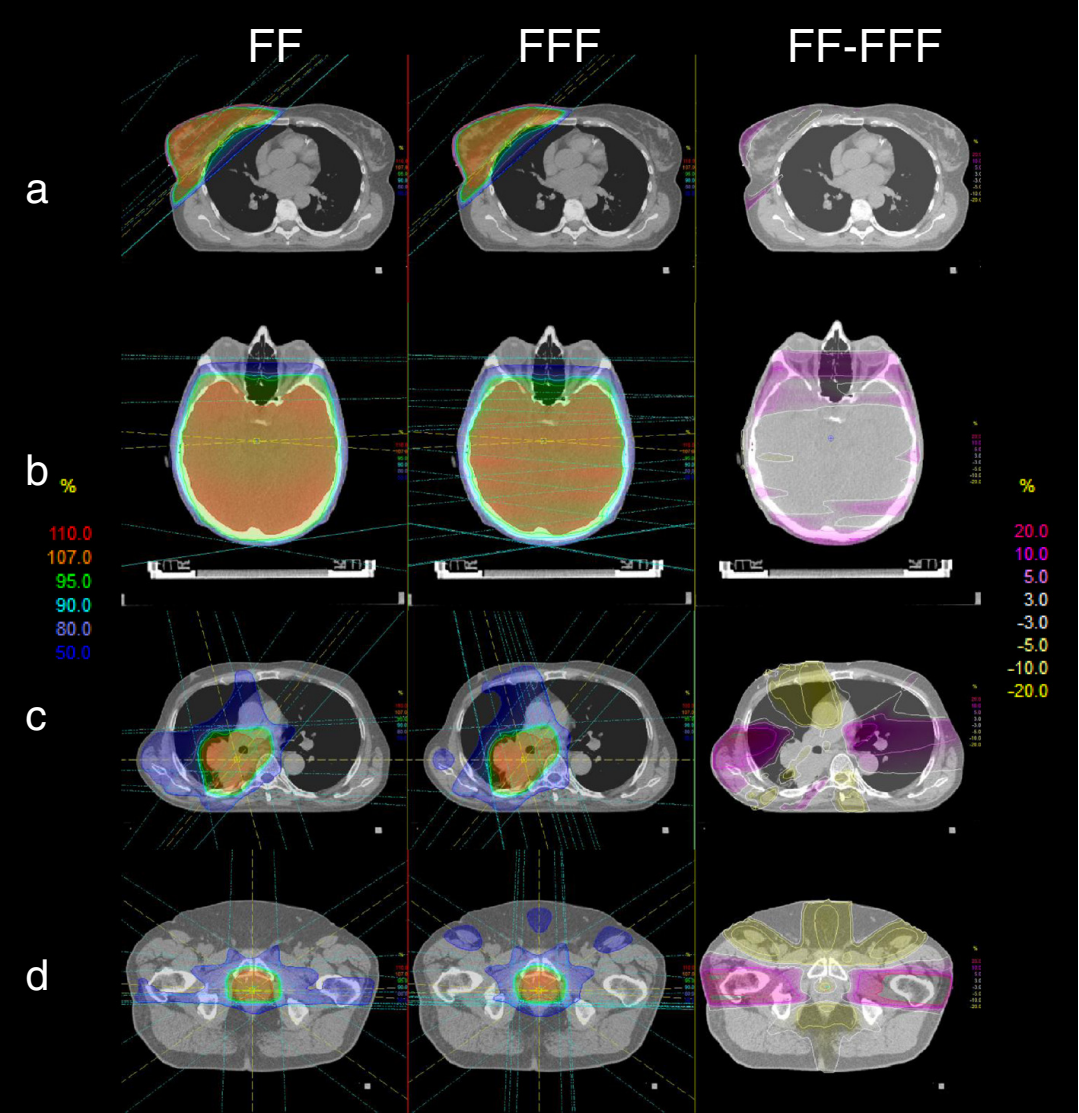
**Exemplary axial dose distributions for four RT locations.** Relative dose distribution at the isocenter plane for: **a** breast, **b** neurocranium, **c** lung, **d** prostate. The left column shows the results of FF plans and the middle column shows the results of FFF plans. The right column shows the relative dose difference FF – FFF. When defining FF plans as the gold standard, yellow isoshades indicate more dose contribution from FFF beams. Purple isoshades indicate more dose contribution from FF beams.

Figure 2 displays the results of typical dose distributions at the isocenter plane for the investigated RT locations. FF plans served as gold standard. When using simple opposing RT plans i.e. breast and neurocranium, the resulting relative dose distributions are very similar. The relative difference dose distributions show deviations below 10% in the majority of the cases. This is the case in particular in the axilla region outside the PTV in the breast group. An effective dose fall off could be better realized with FFF beams.

In the neurocranium group, FFF plans benefit from a significant higher dose fall off between the PTV and the OAR eyes. When the number of initial beams is equal to or greater than three (lung and prostate) the treatment planning is more difficult in terms of achieving the dose objectives with FFF beams. Deviations could be found in the relative difference dose distributions (Figure 2c, d) because different gantry angles were associated with different levels of modulation between FF and corresponding FFF plans.

### Technical parameter analysis

**Figure 3:**
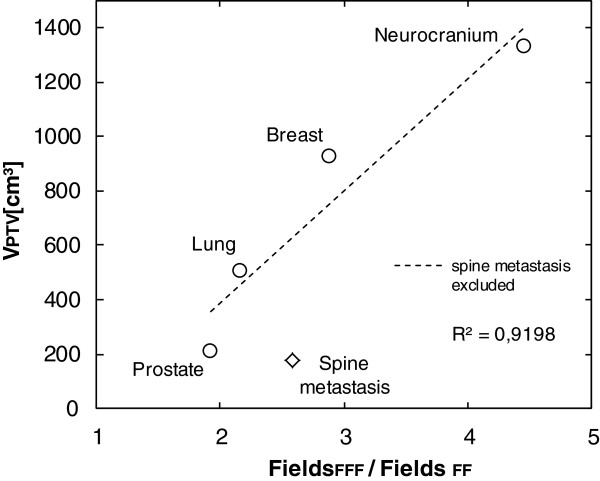
**Diagram correlating PTV volume with the field number coefficient.** Correlation between the mean values for the location-specific coefficient Fields_FFF_/Fields_FF_ and the volume of PTV. The quality of the linear fit is given with R^2^. The data point spine metastasis was excluded due to inconsistent FF planning with virtual wedge fields.

**Figure 4:**
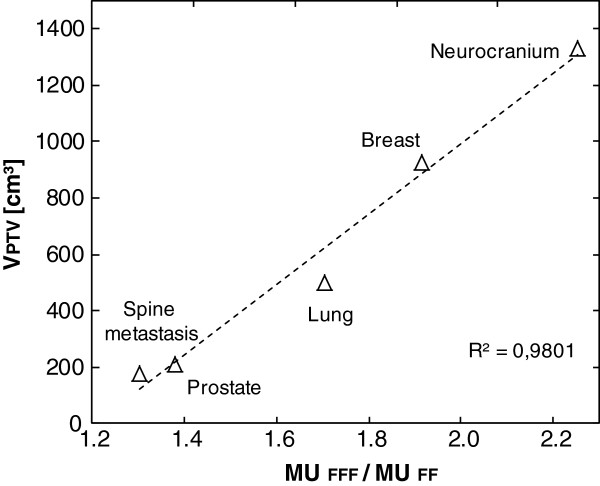
**Diagram correlating PTV volume with MU coefficient.** Correlation between the mean values for the location-specific coefficient MU_FFF_/MU_FF_ and the volume of PTV. The quality of the linear fit is given with R^2^.

Table 6 shows the results of the technical plan parameters. Significantly more fields were necessary (p<0.001) in order to achieve PTV coverage for each RT location when using FFF beams. Figure 3 shows the correlation between the location-dependent ratio of the number of fields (fields FFF/fields FF) and the mean PTV volume. Excluding the spine metastasis location the linear fit correlates with R^2^=0.919. The exclusion of the spine metastasis group was necessary because virtual wedge fields were also permitted for FF plans at the beginning of the studies. The average increase for fields is between 1.9 (prostate) and 4.5 (neurocranium). Significant increases were seen for all groups in the mean number of required MU with the use of FFF beams (p<0.001). Figure 4 shows the correlation (R^2^=0.980) between the location-dependent ratio of the number of MU (MU _FFF_ / MU _FF_) and the average PTV volume. The increases range from 1.3 (spine metastasis) to 2.2 (neurocranium). The lower section of Table 6 shows the results of the radiation times (time from first beam on to last beam off) for six FFF and six FF plans. The additional time required for the application of an FFF plan is on average 68s per fraction for breast, 87s for neurocranium, 60s for lung and 28s for prostate.

**Table 6:** Summary of the technical parameters for treatment plans created with FF and FFF beams

**Parameter**	**Breast**	**Neurocranium**	**Lung**	**Spine metastasis**	**Prostate**
Gantry positions	2	2	3	4	5
Fields	5.2 ± 0.7	3.0 ± 0.0	6.9 ± 2.1	4.1 ± 0.6	5.9 ± 0.9
	**14.8 ± 2.0**	**13.4 ± 1.2**	**14.9 ± 5.1**	**10.6 ± 2.5**	**11.4 ± 1.7**
Range Fields	4 - 6	3 - 3	5 - 10	3 – 5	5 - 7
	**12 - 18**	**11 - 15**	**10 - 25**	**5 – 14**	**9 - 15**
MU	244.3 ± 9.0	318.0 ± 2.3	257.4 ± 13.6	317.8 ± 11.8	305.7 ± 17.8
	**451.8 ± 27.8**	**686.1 ± 39.3**	**427.0 ± 76.5**	**411.5 ± 49.1**	**416.7 ± 33.6**
Range MU	227 - 259	314 - 322	245 - 282	301 – 340	280 - 350
	**393 - 494**	**623 - 738**	**351 - 564**	**346 – 505**	**351 - 485**
tx time [s]	146 ± 5	112 ± 1	128 ± 26	-	165 ± 11
	**213 ± 21**	**199 ± 14**	**188 ± 34**	**-**	**193 ± 21**
Δ FFF – FF [s]	68	87	60	-	28

FFF results are in bold. Statistical significance is defined for p < 0.01. Tx-times for spine metastasis plans are excluded due to inconsistent FF planning with virtual wedge fields.

### Dosimetric analysis

Six FFF plans of each of the studied tumor locations (n=36) were measured with a total of 96 modulated fields (multi static fields) using 2D dosimetry on the ARTISTE in Görlitz. Table 7 shows the results for γ<1 for all studied RT locations. For 96 fields a mean and a standard deviation of 99.7% ± 0.9% was found for γ<1. The mean percentage of γ>1 was below 0.9% for all locations.

**Table 7:** Summary of dosimetric measurements with a 2D detector array

	**All**	**Breast**	**Neurocranium**	**Lung**	**Spine metastasis**	**Prostate**
Modulated fields	96	12	12	18	24	30
γ<1 Mean +SD [%]	99.7 ± 0.9	99.9 ± 0.4	99.1 ± 0.7	100.0 ± 0.0	99.5 ± 2.2	100.0 ± 0.0

The table shows the number of measured multistatic fields, mean and standard deviation for γ<1 with the gamma criteria 3mm DTA (distance-to-agree) and 3% dose difference for doses above 5% of the dose maximum.

## Discussion

This retrospective study aims to clarify whether the quality of 3D conformal RT plans is influenced by the use of FFF beams. The plan quality in relation to the PTV is equivalent when FFF beams are used. Independently of the beam production method chosen it was possible to achieve all planning objectives apart from slight deviations. Significantly more fields were necessary to achieve the required dose in the PTV for FFF plans for all PTV sizes. It was possible to determine a correlation (R^2^=0.919) between PTV volumes in comparison to the additional fields necessary in FF plans. Underdoses to the PTV needed to be compensated by adjusting the leaves in the beams eye view according to the 95% isodose line.

For a typical depth dose of 10 cm a radial dose decrease of approximately 3% per cm must be compensated at 7MV FFF and 5% per cm for 11MV (d=10 cm, SSD=90 cm FS=20 × 20 cm^2^). This procedure led to a slight loss of homogeneity (HI) in the PTV, especially for larger PTVs such as breast or neurocranium. For smaller PTVs such as prostate with field sizes < 7 × 7 cm^2^ a quasi-plateau favours radial compensation with additional fields. The radial dose loss at 10 cm depth is a maximum of 10% for 11MV. On average 1.9 times more fields were necessary in order to achieve the planning requirements. Across all RT locations additional time requirements of up to 20 minutes were determined for treatment planning when using FFF beams. This extra time is solely based on the work for the manual shaping of the additional fields. The adaptation of additional fields was primarily oriented to isodoses in the beam’s eye view. Smaller field sizes were normally necessary in the course of the planning process in order to achieve dose conformity. The smallest field size used in this study was 3 × 3 cm^2^.

It was also possible to prove a clear correlation (R^2^=0.980) between the mean PTV volume and the increase factor of required monitor units when using FFF beams. An increase in PTV size of 100 ccm^3^ increases this factor by approximately 7%. For the neurocranium group (largest PTV) the MU was seen to double (2.2 times) on average. For small volumes such as prostate an average of 30% more MU was required. In breast treatment, favourable FFF plans could be generated as described by Mah [15]. If the isocenter was located on the thorax wall then the FFF profile favoured lateral fit to the breast. In the breast group an average of 14.8 ± 2.0 fields were necessary.

Despite the increased number of fields and MU for FFF plans no relevant differences were found for the low dose parameters V5Gy and V10Gy. One possible reason for this could be the partial compensation from the reduced treatment head leakage described by various authors and scattered radiation in FFF [1,12]. Kragl et al. presented dosimetric measurements that revealed a reduction in low dose exposure outside the field from peripheral doses, especially when using lower FFF energy (6MV) [6]. It is at least questionable whether these effects can be modelled correctly in the planning system and this was not investigated in the course of this study using dose measurements.

It was possible to comply with all restrictions in relation to the studied OAR in the planning of both FF and FFF beams. It is probable that the effects of reduced low dose exposure led to a significantly lowered mean lung dose for contra-lateral lung in the breast group. Howell et al. reported about errors in the dose calculations in this context, even 3 cm from the edge of the treatment field. They found underestimated out-of-field-doses that averaged 40% through the TPS [16]. In this study, the OAR contralateral lung and heart (breast group) are particularly affected. Most of these OAR were not penetrated by the treatment beam and absolute dose values should be evaluated with caution. Although there was also a slightly significant reduction in the ipsilateral lung one can assume that the strong scatter from the PTV completely overshadowed the influence of the reduced low dose exposure outside the field with FFF [5]. In the prostate group the mean and the V50Gy dose for rectum and bladder were slightly but not significantly increased in FFF plans. Despite the formation of a quasi-flat beam profile at field sizes < 7 × 7 cm^2^, this group showed that additional fields that increase PTV coverage were also necessary, on average multiplied by 1.9. A significantly increased mean heart dose of 0.8Gy for FFF plans in the lung group can be ascribed to increased dose modulation (fields) of beam direction via the heart. In this case it would be necessary to use additional fields for the ventral or dorsal beam direction because of the inhomogeneity step to the lung, particularly with hilar enlargement of the mediastinal PTV on the left side. However, a minimum field size is necessary in order to retain the lateral secondary electron equilibrium.

The average additional time that is required for the application of a fraction in FFF mode is between 28s for a small PTV (prostate) and 87s for a large PTV such as the neurocranium. The timesaving effect of the high dose performance of 2000 MU/min at 7MV_FF_ and 11MV_FFF_ was completely neutralized because of the additional fields that were required. In order to satisfy the beam formation time in FFF mode the dose rate is automatically reduced to 500 MU/min when 10 MU or less is selected. It was not possible to carry out the timesaving bundling of individual fields with the same energy and the same gantry and collimator angles for one IMRT sequence. As FiF techniques are common in everyday clinical use, sequencing in the planning system for RT is recommended in order to save more time. It could be possible to generate time savings during the planning process through the use of plan libraries.

Several authors reported increased surface dose when using FFF beams [1]. Based on DVH analysis this fact could not be proven in this study. In the breast group analysis of the full PTV (without 3 mm distance from the external) no significant differences were found in any studied parameter. The question of the correct modelling in the planning system must also be posed here.

The relative energy spectrums between 6MV_FF_ and 7MV_FFF_ in MasterPlan display a slight shift to a greater mean energy with FFF (2.4MV versus 2.7MV for 6MV_FF_ and 7MV_FFF_, data not shown). This data displays good correlation with studies by Dzierma et al. for the spectrum definition in a pinnacle treatment planning system (2.2MV versus 2.5MV for 6MV_FF_ and 7MV_FFF_) [12]. As the ARTISTE beam line was not changed except for the flattening filter this seems to indicate that a slight increase in energy has taken place. Wang et al. describe an increased surface dose with 6MV_FFF_ und 10MV_FFF_ on a Varian TrueBeam linear accelerator that is proven by measurement but not clinically relevant. [17].

3D-CRT FFF plans that were generated in this study were based on the same beam direction (field setup) as FF plans that are used clinically. In all study groups additional fields that compensate for radial weakening lead to increases of 1.3 to 2.2 times in the number of MU. Hall estimates the influence on the risk of radiation-induced malignancy because of the MU increase for modulated FF fields to be an additional 0.25% [18]. On the other hand, the removal of the flattening filter as a major source of scatter has a beneficial effect on treatment head leakage and thus on the peripheral dose. This enabled Kragl et al. to prove a reduction in peripheral dose of 16% (6MV) or 18% (10MV) [11] using modulated FFF beams on an Elekta accelerator.

The FFF beam model that is implemented in MasterPlan (eCC) for 7MV and 11MV was able to calculate a correct 2D dose prediction. Over 96 multi static field measurements the mean for γ<1 was found to be 99.7% ± 0.9%. The determined values show good correlation with the studies by Kragl et al. for the use of modulated fields [11]. No clear correlation could be determined between the average volume of the RT location and the mean gamma failure rate (γ>1). The measurements showed that the dose calculation not only works for small field sizes (prostate) around the central beam in the area of a quasi-dose plateau, but also in expansive and extremely peripheral fields or parts of fields (neurocranium). The radial reduction in dose caused by FFF is up to 25% for neurocranium plans at a depth of 10 cm at 11MV. The average proportion of γ<1 was lowest here at 99.1% ± 0.7% but is highly acceptable.

## Conclusions

This study has shown that the exclusive use of a linear accelerator in FFF mode is feasible in 3D CRT. It was possible to realize FFF plans of comparable quality to conventional FF plans for typical radiotherapy treatment locations. It was possible to adapt radial weakening of the fluence in FFF mode optimally to the given PTV using the FiF planning methods that are standard in clinical practice. The 2D dosimetric validation of the modulated fields revealed correct reproduction of the characteristic FFF parameters in the treatment planning system. It was not possible to compensate for the additional time required for the necessary additional fields in FFF plans despite the dose rate being up to six times higher in FFF mode.

## Competing interests

The Department of Radiation Therapy and Radiooncology at the Radiologische Allianz Hamburg receives research grants from Nucletron/ELEKTA, Siemens AG Healthcare sector and Prowess Inc.

## Authors’ contributions

MK and MS contributed significantly to study design and concept. MK, AB, SH, MS and BG were responsible for treatment planning and dose measurements. Data analysis and interpretation of results were performed by MK. Corrections and/or improvements were suggested by MS, SH and FW. FW was responsible for PTV contouring and clinical evaluation of the treatment plans. All authors read and approved the final manuscript. The authors like to thank Gerald Reinig from Siemens AG for helpful comments.
